# Modification of Octavinyl POSS and Its Effect on the Mechanical Properties and Thermal Stability of Silicone Rubber/POSS Composites

**DOI:** 10.3390/nano15221706

**Published:** 2025-11-12

**Authors:** Junjie Peng, Yong Zhang

**Affiliations:** Shanghai Key Laboratory of Electrical Insulation and Thermal Aging, School of Chemistry and Chemical Engineering, Shanghai Jiao Tong University, Shanghai 200240, China

**Keywords:** silicone rubber, multifunctional POSS, mechanical properties, thermal stability

## Abstract

Octavinyl polyhedral oligomeric silsesquioxane (POSS) can be used to improve the thermal stability of silicone rubber (SR). However, POSS nanoparticles tend to agglomerate in SR matrix, negatively affecting the reinforcement role of POSS for SR, and consequently limiting the practical application of SR/POSS composite. To address the issue, multifunctional POSS (m-POSS) was synthesized via a thiol-ene click reaction and used as a novel heat-resistant filler for SR. The results demonstrate that m-POSS containing both vinyl and siloxane groups was successfully synthesized, with the main product exhibiting a molecular weight of approximately 1587 g mol^−1^. At the POSS loading of 1.5 phr, SR/m-POSS (100/1.5) composite has much better mechanical properties and thermal stability than SR/POSS (100/1.5) composite. With increasing m-POSS loading from 1.5 to 4.5 phr, the thermal stability of SR/m-POSS becomes better, while the tensile strength decreases. SR composite filled with 1.5 phr m-POSS has an excellent balance in thermal stability and mechanical properties, with a tensile strength of 9.2 MPa and an elongation at break of 587%. To fill multifunctional polyhedral oligomeric silsesquioxane containing vinyl and siloxane groups into SR is an effective approach to producing composites with excellent properties.

## 1. Introduction

Silicone rubber (SR) has excellent thermal stability and extensive uses in complex high-temperature applications, particularly in the aerospace and automotive industries [1,2,3]. To further extend its service life in high-temperature environment, some heat-resistant fillers are filled in SR to enhance its thermal stability [4,5,6]. However, the overloading of the fillers leads to filler agglomeration, undermining the mechanical properties and durability of SR [7,8,9]. Thus, it is of great practical research value to develop an effective approach to produce SR composites with excellent properties.

Polyhedral oligomeric silsesquioxane (POSS) is an important organic-inorganic nanomaterial well-known for its effectiveness in enhancing the thermal stability and environmental resistance of SR [10,11,12]. However, the agglomeration of POSS and its poor compatibility with polymer will bring about the distortion of mechanical properties [13,14,15,16]. When POSS is not uniformly dispersed at the nanoscale but instead forms microscale aggregates within the polymer matrix, these regions act as stress concentration sites, resulting in a significant decline in the overall mechanical performance of the composites. To solve these problems, Ma et al. [17] employed tetravinyl polysilsesquioxane as a crosslinking modifier in room-temperature vulcanized (RTV) silicone rubber to improve the comprehensive properties. The initial decomposition temperature of the RTV silicone rubber containing 1 phr of tetravinyl polysilsesquioxane increased by 20 °C, although most published studies focused on RTV silicone rubber, with limited attention to high-temperature vulcanized (HTV) silicone rubber. From a practical perspective, HTV silicone rubber can be used in high-temperature, complex, and stringent environments such as aerospace and aviation, requiring good comprehensive properties [18,19,20]. However, a few studies include how multifunctional POSS influences the mechanical properties of HTV silicone rubber. Meng et al. [21] improved the thermal stability of HTV silicone rubber by adding some allyl isobutyl-POSS, highlighting the potential of multifunctional POSS to enhance the performance of SR. The effect of multifunctional POSS on the mechanical properties of HTV silicone rubber needs further investigation. In our previous work, SR was filled with low-content POSS and carbon nanotube, and the resultant SR composites had good mechanical properties and enhanced thermal stability [22]. When 0.1 phr of POSS and 1 phr of CNTs are added simultaneously, the tensile strength and elongation at break of the SR composite are 10.2 MPa and 487%, respectively, and the initial decomposition temperature is 458 °C. To further improve the properties of SR, constructing multiple network structures using high-content multifunctional POSS in SR might be a feasible strategy to achieve a good combination of various properties.

To meet the practical demands of SR, silica is often incorporated in SR to enhance its mechanical properties [23,24,25]. However, the presence of active silanol groups on the surface of silica can be problematic [26,27,28]. At elevated temperatures, silica hydroxyl groups may cause the breakdown of silica-oxygen bonds, initiating the degradation of SR, and reducing the thermal stability of SR composites [29]. Additionally, these active silanol groups can cause the fillers to agglomerate in the matrix, further degrading the properties of SR composite [30,31]. To address the issues, Dong et al. [32] used *γ*-aminopropyl triethoxysilane-modified silica as a crosslinking agent for polysiloxanes containing *γ*-chloropropyl groups and thus prepared SR composites with excellent properties. Inspired by the above study results, we synthesized multifunctional POSS that could interact with both the macromolecular chains and the fillers in SR composites, aiming to prepare SR composites with excellent properties.

In this work, multifunctional polyhedral oligomeric silsesquioxane (m-POSS) was synthesized by the thiol-ene click reaction and used as a novel heat-resistant filler for SR composites. The carbon-carbon double bonds in m-POSS could chemically react with SR macromolecules through the free radical reaction initiated by an organic peroxide. The ethoxy groups of m-POSS could react with the silanol groups of silica, thus improving the dispersion of silica in SR. The changes in the physical state of POSS before and after the modification, as well as the relationship between the crosslinking structure and the properties of the composites, were systematically studied. Consequently, the preparation of multifunctional POSS and its incorporation into SR composites provides an effective strategy for obtaining SR composites with excellent properties.

## 2. Experimental

### 2.1. Materials

Silicone rubber (SR, *M*n = 5.9 × 10^5^, containing 40 phr silica) and 2,5-dimethyl-2,5-di(*tert*-butylperoxyl)hexane (DBPMH) were produced by Midgold Fine High-Tech Materials (Shenzhen, China) Company. Concentrated hydrochloric acid 37% (AR), toluene, 3-Mercaptopropyltriethoxysilane (KH580), azobisisobutyronitrile, and acetone were purchased from Shanghai Titan Technology (Shanghai, China) Company. Octavinyl polyhedral oligomeric silsesquioxane (POSS) was synthesized in our laboratory.

### 2.2. Synthesis of Multifunctional Polyhedral Oligomeric Silsesquioxane (m-POSS)

**Figure 1:**
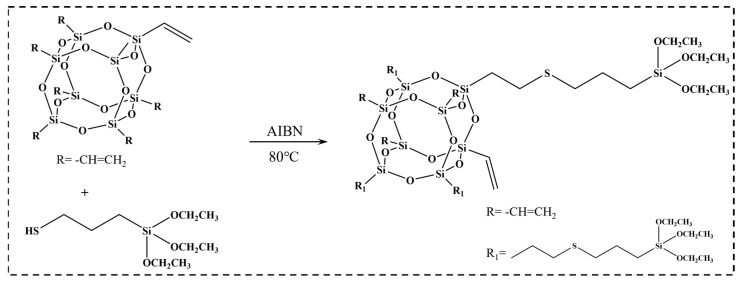
Synthetic reaction of m-POSS.

Based on the methodology proposed by Zheng et al. [33], m-POSS was synthesized via the thiol-ene click reaction, as shown in Figure 1. Initially, 2.5 g octavinyl polyhedral oligomeric silsesquioxane, 3.76 g 3-mercaptopropyltriethoxysilane, 40 mL toluene, and 0.02 g azobisisobutyronitrile were added in a three-necked flask (500 mL). Under nitrogen gas atmosphere, the solution was refluxed at 80 °C for 3 h. After filtering and drying to remove the solvent, 5.2 g m-POSS was obtained with a yield of 83%.

### 2.3. Preparation of SR/m-POSS Composites

The preparation and vulcanization procedures for SR composites in this chapter are based on the methods proposed by Peng et al. [22], without significant modifications. Basic formulation was SR (containing 40 phr silica) 100, DBPMH 2, POSS 1.5 phr or m-POSS variable (0, 1.5, 2.5, 3.5, and 4.5 phr). Firstly, SR and POSS or m-POSS were mixed at 60 °C for 10 min. The resultant compound was further mixed at 150 °C for 3 min. Then, 2 phr DBPMH was added in the compound on a two-roll at 25 °C for 5 min. The compound was cured at 10 MPa and 170 °C, with optimum time *t*_90_ plus 3 min.

### 2.4. Characterizations

The curing characteristics of SR composites were determined by RPA2000 rubber process analyzer (TA Instruments, New Castle, NJ, USA) at 170 °C and a test frequency of 1.7 Hz. The ^1^H and ^29^Si NMR spectra of m-POSS were obtained on a Bruker AVANCE III 400 MHz spectrometer using CDCl_3_ as the solvent. Fourier transform infrared spectroscopy (FTIR Thermon Fisher Scientific, Waltham, MA**,** USA) was employed to characterize the chemical structure of m-POSS in the wavenumber range of 4000–450 cm^−1^, with a resolution of 4 cm^−1^ and 16 scans accumulated. Matrix-assisted laser desorption/ionization time of flight mass spectrometry (MALDI-TOF MS, BRUKER, Beverly Hills, MA, USA) was employed to determine the molecular weight of m-POSS. 2,5-Dihydroxybenzoic acid (DCTB) was used as the matrix, and the sample was dissolved in toluene at a concentration of 10 mg/mL. The sample solution, matrix, and silver salt were mixed at a volume ratio of 5:25:1 and homogenized using a vortex mixer. Subsequently, 1 μL of the mixture was deposited onto the target plate with a pipette and allowed to dry at room temperature prior to analysis. The SR composites were fractured in liquid nitrogen, and the fracture surfaces were sputter-coated with gold for 30 s. The microstructure of the composites was subsequently examined using a scanning electron microscope (SEM, S-2150, Hitachi High-Technologies, Tokyo, Japan). The SR composites were cryo-ultramicrotomed in liquid nitrogen using an ultramicrotome (UC6, Leica Microsystems GmbH, Weitzlauer, Germany), and the resulting ultrathin sections were subsequently examined by transmission electron microscopy (TEM, JEM-2100, JEOL Ltd., Tokyo, Japan). Thermal stability of SR composites was evaluated using thermogravimetric analysis (TGA, TA Instruments, New Castle, NJ, USA) under a nitrogen atmosphere with a heating rate of 20 °C/min and a temperature range of 50–800 °C. Tensile properties of SR composites were evaluated on the same Instron 3365 system at a tensile rate of 500 mm min^−1^ using dumbbell-shaped specimens with dimensions of 75 mm × 4 mm × 1 mm (length × width × thickness). Five valid tests were performed for each sample, and the mean value was reported.

## 3. Results and Discussion

### 3.1. Characterization of m-POSS

**Figure 2:**
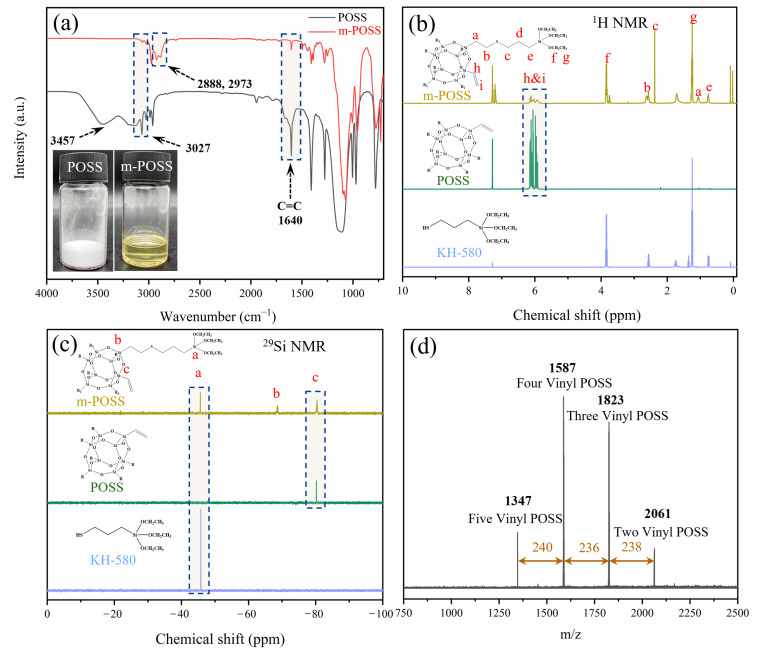
(**a**) FTIR spectra, (**b**) ^1^H-NMR, (**c**) ^29^Si-NMR, and (**d**) MALDI-TOF MS spectrum of m-POSS.

First, m-POSS was prepared through the reaction between POSS and KH580, and characterized by FTIR and NMR. In Figure 2a, POSS is a white crystalline powder, while m-POSS is a yellow liquid, indicating that the chemical modification of POSS with KH580 changed the physical state of POSS. Notably, the broad absorption peak observed near 3457 cm^−1^ in the FTIR spectrum of POSS is attributed to the O-H stretching vibration of trace moisture present in the KBr pellet, rather than to hydroxyl groups in the sample itself. In addition, based on the initial feed ratio, a certain amount of vinyl groups should remain in the molecular structure of POSS. A small stretching vibrations peak at 1640 cm^−1^ and 3027 cm^−1^ is observed in the FTIR spectrum of m-POSS (Figure 2a), corresponding to the characteristic absorptions of vinyl groups. This result indicates the presence of a small amount of C=C bonds in m-POSS. Meanwhile, new stretching vibrations peak appears at 2888 cm^−1^ and 2937 cm^−1^, which are attributed to the methylene group stretching vibrations by the grafting of KH580. These characteristic peaks confirm that KH580 reacts with POSS through a thiol-ene addition reaction, leading to the consumption of double bonds and the successful incorporation of alkyl chains into the POSS framework. Additionally, the ^1^H NMR spectra are shown in Figure 2b, and multiple proton signals appeared from 5.8 to 6.2 ppm, confirming the presence of vinyl groups in the m-POSS. Furthermore, the ratio of the attributed proton areas, a:(b + c):d:e:f:g:(h + i) ≈ 1:2:1:1:3:4.5:1.5, indicating that about half of the vinyl groups in POSS have taken part in the reaction with KH580. The ^29^Si NMR spectra are shown in Figure 2c; there was a new chemical shift that appeared at −68.6 ppm, demonstrating the introduction of siloxane groups in m-POSS. These experimental results collectively affirm the successful synthesis of m-POSS.

Suppose that four vinyl groups in a POSS molecule reacted with four KH580 molecules, the theoretical molecular weight of m-POSS should be 1587 g/mol. In Figure 2d, MALDI-TOF MS spectrum of m-POSS has four characteristic peaks at 1347, 1587, 1823, and 2061 g/mol. Notably, m-POSS exhibits a main molecular weight of 1587 g/mol, indicating that m-POSS with the intended molecular structure was obtained as we wished.

### 3.2. Curing Characteristics of SR/m-POSS Composites

**Figure 3:**
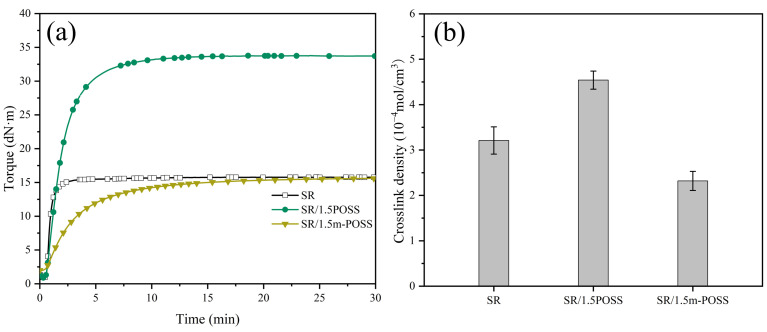
(**a**) Curing curves and (**b**) crosslink density of SR composites (*n* = 3).

Many carbon-carbon double-bond groups of m-POSS could be grafted to the macromolecular chains of SR through the chemical reaction initiated by organic peroxide. The curing characteristics of SR/m-POSS composite were studied by RPA2000. The addition of POSS or m-POSS increases the curing safety of the composite but decreases its curing rate (Figure 3a and Table 1). Notably, the maximum torque (*M*_H_) value significantly increases after 1.5 phr POSS was added to SR. In contrast, the addition of 1.5 phr m-POSS results in only a slight decrease in *M*_H_.

Figure 3b shows the crosslink density of composites. Upon adding 1.5 phr POSS, the crosslink density of vulcanized SR composites increases. This is due to the chemical crosslinking reaction of the carbon-carbon double bonds in POSS with the double bonds of SR initiated by organic peroxide, leading to the formation of rigid crosslink sites. However, upon adding 1.5 phr m-POSS, the crosslink density of the vulcanized SR composites decreases. It could be related to the reduced content of double bonds in the m-POSS structure. Consequently, m-POSS tends to chemically graft onto the SR molecular chains rather than participate in crosslinking reactions, which consumes more organic peroxides, and ultimately leads to a further decrease in the overall crosslink density of SR composites.

**Table 1:** Curing data of silicone rubber composites.

Material	*t*_10_/min	*t*_90_/min	*M*_H_/dN·m	*M*_L_/dN·m	*M*_H_-*M*_L_/dN·m
SR	0.2	1.68	15.8	0.91	14.9
SR/1.5POSS	0.3	4.98	33.8	0.89	32.9
SR/1.5m-POSS	0.3	10.2	15.2	1.24	13.9

### 3.3. Mechanical Properties of SR/m-POSS Composites

**Figure 4:**
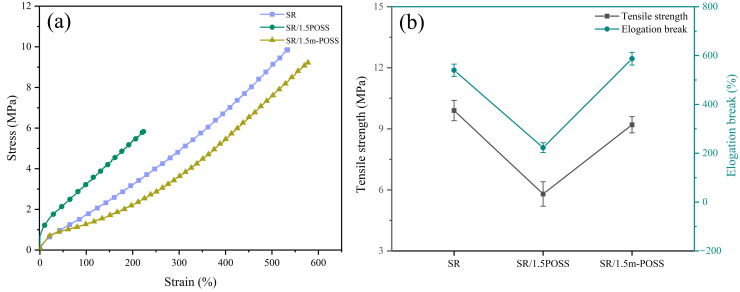
(**a**) Stress–strain curves and (**b**) mechanical properties of SR composites.

Figure 4a shows the stress–strain curves of SR, SR/POSS (100/1.5), and SR/m-POSS (100/1.5) composites. When 1.5 phr of POSS was added to SR, the tensile strength of SR/POSS (100/1.5) composite decreased compared with SR composite, reaching 5.8 MPa. However, SR/m-POSS (100/1.5) composite still retained good mechanical properties, exhibiting the tensile strength of 9.2 MPa and elongation at break of 587% (Figure 4b), which are much higher than that of SR/POSS (100/1.5) composite, with the increases of 60% and 160%, respectively. To assess whether the observed differences in mechanical performance were statistically significant, we compared the SR/POSS and SR/m-POSS composites using an unpaired two-sample (two-tailed) Student’s *t*-test. As summarized in Table 2, SR/m-POSS showed higher tensile strength and elongation at break than SR/POSS composite, with *p* < 0.001 for both properties, indicating a highly significant advantage for SR/m-POSS composite. The significant increase in the tensile strength of SR/m-POSS (100/1.5) composite is likely due to the fulfillment of good dispersion of m-POSS in SR matrix. Furthermore, as shown in Table 3, the SR/m-POSS composite still exhibits good mechanical properties compared to the previously reported SR composite.

**Figure 5:**
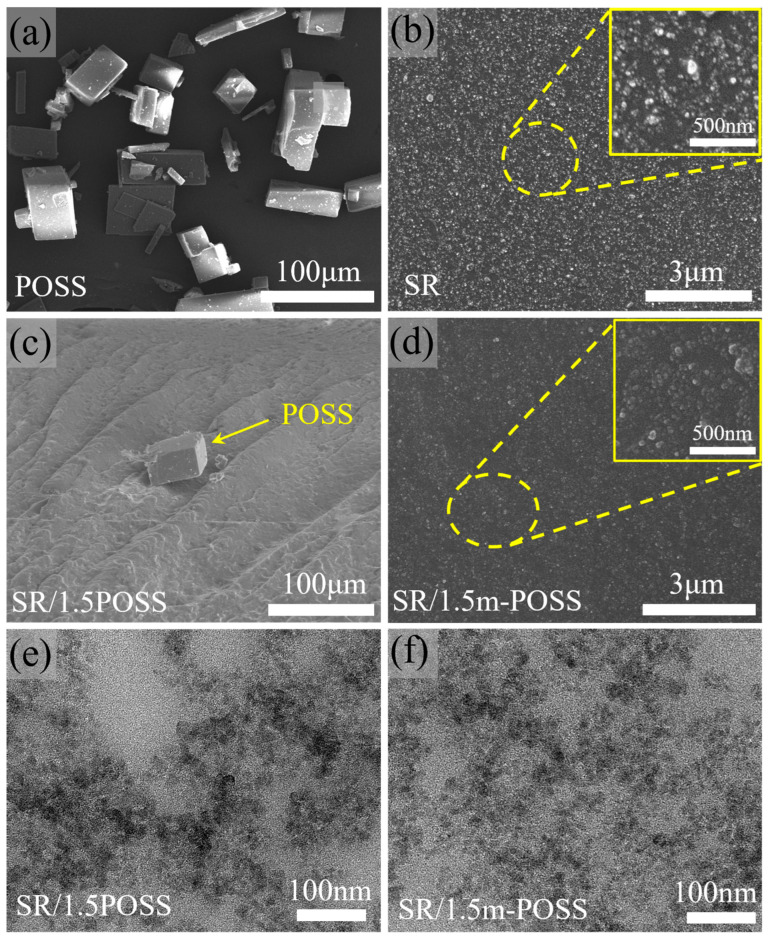
SEM images of (**a**) POSS, (**b**) SR, (**c**) SR/POSS (100/1.5), and (**d**) SR/m-POSS (100/1.5). TEM images of the cross-section of (**e**) SR/POSS (100/1.5) and (**f**) SR/m-POSS (100/1.5) composites.

The morphologies of POSS, SR, SR/POSS (100/1.5), and SR/m-POSS (100/1.5) composites were observed by SEM. POSS particles exhibit a regular cubic morphology with the particle sizes ranging from 40 to 80 μm due to the aggregation (Figure 5a). Figure 5b shows that the fillers are uniformly dispersed in SR composite. However, when 1.5 phr POSS was incorporated into SR, a significant aggregation of POSS was observed, accompanied by the presence of voids between POSS crystals and SR matrix (Figure 5c). The poor mechanical properties of SR/POSS (100/1.5) composite could be attributed to the aggregation of POSS particles with sizes up to approximately 20 μm, which hinders their uniform nanoscale dispersion within the SR matrix. In contrast, m-POSS could not be seen in the fracture cross-section of SR/m-POSS (100/1.5) composite, as shown in Figure 5d, indicating that nanoscale dispersion was achieved in the matrix. This uniform nanoscale dispersion contributes significantly to the enhanced mechanical properties of the composite. Additionally, the dispersion of silica in SR/POSS (100/1.5) and SR/m-POSS (100/1.5) composites were examined by TEM. It can be clearly observed that SiO_2_ particles are more uniformly dispersed in SR/m-POSS (100/1.5) composite (Figure 5e,f). This should be attributed to the interaction between the ethoxy groups in m-POSS and the silanol groups of SiO_2_, which could promote the dispersion of silica within the matrix.

### 3.4. Thermal Stability of SR/m-POSS Composites

**Figure 6:**
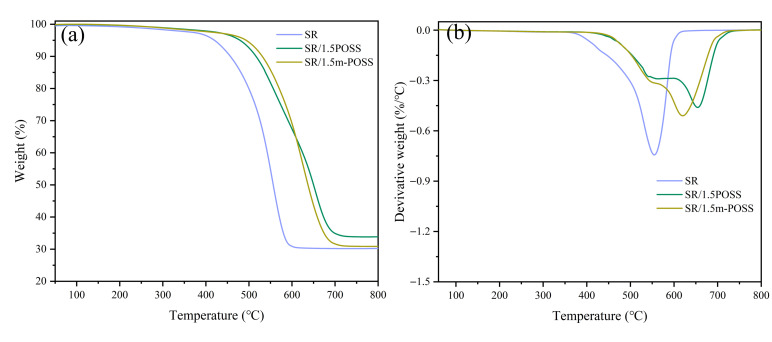
(**a**) TGA curves and (**b**) differential thermal gravity curves of SR composites.

The addition of POSS in polymers is a successful strategy for enhancing the thermal stability of polymers. It is crucial to study the influence of m-POSS on the thermal stability of SR by TGA. The temperature at 5% weight loss (*T*_5%_) for SR, SR/POSS (100/1.5), and SR/m-POSS (100/1.5) is 419, 484, and 494 °C, respectively (Figure 6a and Table 4). The maximum decomposition temperature (*T*_max_) of SR/m-POSS (100/1.5) reaches 620 °C, much higher than 552 °C for SR (Figure 6b). During the initial thermal decomposition of SR under an inert atmosphere, depolymerization is the dominant process, producing cyclic oligomers along with small amounts of linear oligomers. As the temperature increases, the crosslinking structure of SR undergoes cleavage and structural rearrangement, and the final residue is mainly composed of inorganic fillers. These results clearly demonstrate that adding m-POSS significantly enhances the thermal stability of SR.

In general, a high crosslink density in SR favors high thermal stability, as a denser crosslinked network restricts the movement of molecular chains. Surprisingly, despite SR/POSS (100/1.5) composite has high crosslink density than SR/m-POSS (100/1.5) composite (Figure 3b), SR/POSS (100/1.5) composite still exhibits lower thermal stability. To further investigate the influence of m-POSS on the thermal stability of SR, the number of POSS cage structures within the crosslinked network of both composites was quantified. At a given weight of POSS, the number of the cage structure (POSS molecule) of SR/POSS (100/1.5) composite is 2.4 times higher than that of SR/m-POSS (100/1.5) composite. This difference in the number of the cage structure and the difference in crosslink density influences the thermal stability of SR, but the well dispersion of m-POSS should be a more important factor affecting the thermal stability of SR. This is because m-POSS can enter SR crosslink network through chemical reactions; then, the movement of SR macromolecular chains could be restricted, thus enhancing the thermal stability of SR. Therefore, these interactions account for the better thermal stability of SR/m-POSS (100/1.5) than SR/POSS (100/1.5) composite.

**Table 4:** TGA data of SR composites in N_2._

Materials	*T*_5%_ (°C)	*T*_10%_ (°C)	*T*_max_ (°C)	Total Mass Loss (%)	Residue at 800 °C (%)
SR	419	456	552	69.9	30.1
SR/1.5POSS	480	516	654	69.1	30.9
SR/1.5m-POSS	494	531	620	66.2	33.8

### 3.5. Effect of m-POSS Content on Properties of SR

**Figure 7:**
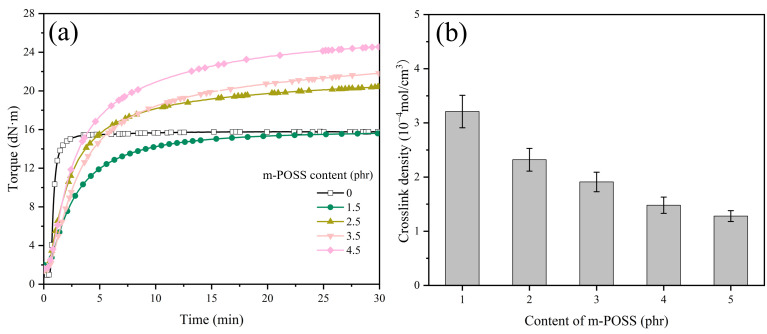
(**a**) Curing curves and (**b**) crosslink density of SR composites with different m-POSS content (*n* = 3).

The effect of m-POSS content on the curing characteristics of SR was studied by RPA2000. When there is an increase in m-POSS content, the *M*_H_ and vulcanization time (*t*_90_) of SR compounds increase (Figure 7a and Table 5). Conversely, the crosslink density of vulcanized SR/m-POSS composites decreases with increasing m-POSS content (Figure 7b). This trend is primarily caused by the participation of m-POSS in the chemical reactions that consume some organic peroxide molecules, leading to a decrease in crosslink density. Additionally, the increase in *M*_H_ of the SR composite may be due to the reaction between the ethoxy groups in m-POSS and the silanol groups of SiO_2_, which promotes the dispersion of m-POSS in SR matrix.

**Table 5:** Curing data of SR composites with different m-POSS content.

m-POSS Content (phr)	*t*_10_/min	*t*_90_/min	*M*_H_/dN·m	*M*_L_/dN·m	*M*_H_-*M*_L_/dN·m
0	0.2	1.68	15.8	0.91	14.9
1.5	0.3	10.2	15.2	1.24	13.9
2.5	0.5	11.24	20.42	1.37	19.1
3.5	0.6	13.93	21.83	1.38	20.6
4.5	0.6	14.21	24.59	1.46	23.1

**Figure 8:**
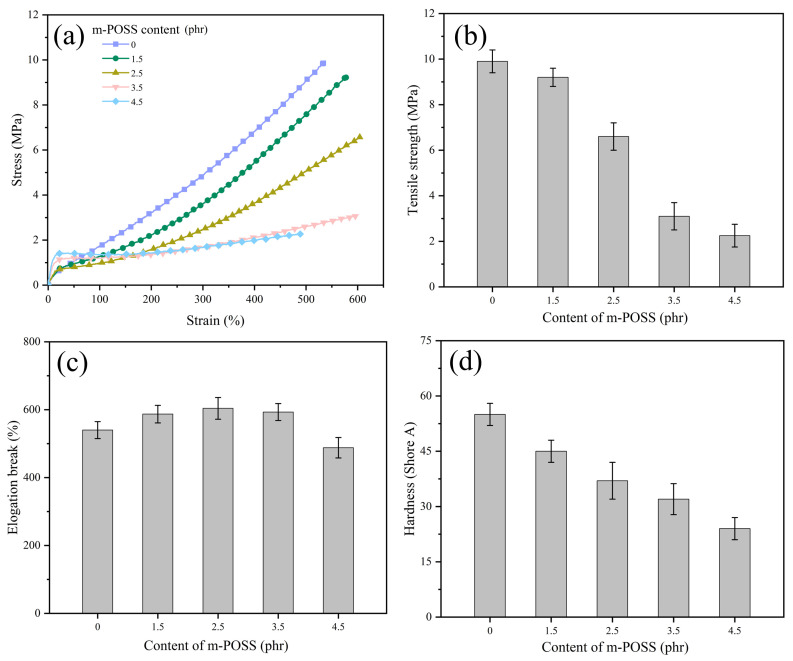
(**a**) Stress–strain curves and (**b**–**d**) mechanical properties of SR composites with different m-POSS content.

Figure 8 shows the mechanical properties of SR/m-POSS composites at different m-POSS contents. When 1.5 phr of m-POSS was added to SR, SR/m-POSS (100/1.5) composite had a tensile strength of 9.2 MPa, showing better mechanical properties than other SR/m-POSS (100/variable) composites. With the further increase in the m-POSS content, the tensile strength and hardness of SR/m-POSS composites decrease, while the elongation at break increases, as shown in Figure 8b–d. This trend is primarily due to the excessive consumption of organic peroxide with the m-POSS content increasing, leading to the disruption of the crosslink network structure and consequently deteriorating the mechanical properties.

**Figure 9:**
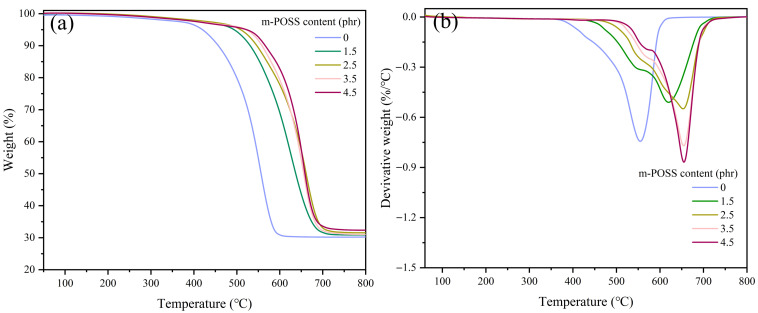
(**a**) TGA curve and (**b**) differential thermal gravity curve of SR composites with different m-POSS content.

The dependence of the thermal stability of SR/m-POSS on the m-POSS content was analyzed by TGA, and the results are shown in Figure 9 and Table 6. When the m-POSS content increased from 0 to 4.5 phr, the thermal stability of the SR/m-POSS composites became much better. The *T*_5%_ of SR/m-POSS (100/1.5) composite is 494 °C, which is 75 °C higher than that of SR composite. This improvement in thermal stability is primarily attributed to two main factors. First, the incorporation of m-POSS into the SR molecular chains introduces a rigid cage structure that restricts chain mobility, thereby retarding the thermal degradation of SR. Secondly, excess hydroxyl groups on the surface of SiO_2_ may undergo condensation reactions with SR chains, leading to the cleavage of the molecular chains [34]. Furthermore, at elevated temperatures, these hydroxyl groups may generate acidic species, which can further accelerate the cleavage or rearrangement of the crosslinking structure of SR, ultimately reducing its thermal stability [35,36]. Therefore, the chemical reaction between the ethoxy groups of m-POSS and the silanol groups on SiO_2_ reduces the hydroxyl content on the filler surface, effectively suppressing this degradation pathway and significantly enhancing the thermal stability of the SR composites.

**Table 6:** TGA data for SR with different m-POSS content in N_2_.

m-POSS Content (phr)	*T*_5%_ (°C)	*T*_10%_ (°C)	*T*_max_ (°C)	Total Mass Loss (%)	Residue at 800 °C (%)
0	419	456	552	69.9	30.1
1.5	494	531	620	69.1	30.9
2.5	509	546	653	68.5	31.5
3.5	523	558	654	68.7	31.3
4.5	525	565	655	67.7	32.3

## 4. Conclusions

Polyhedral oligomeric silsesquioxane containing vinyl and siloxane groups (m-POSS) was synthesized and used as a novel heat-resistant filler to incorporate in SR, obtaining composites with good mechanical properties and thermal stability. The m-POSS was prepared via a thiol-ene click reaction between octavinyl polyhedral oligomeric silsesquioxane and 3-mercaptopropyltriethoxysilane, yielding a product with predominant molecular weight of approximately 1587 g/mol. This thiol-ene click reaction changes the physical state of POSS from solid to liquid, thereby reducing the tendency of POSS to aggregate and promoting its dispersion in SR. Furthermore, the incorporation of m-POSS effectively promotes the dispersion of silica in SR composite.

At the POSS loading of 1.5 phr, SR/m-POSS (100/1.5) composite has better mechanical properties and thermal stability than SR/POSS (100/1.5) composite. The m-POSS content significantly affects the crosslink density of SR composite. With increasing m-POSS content, the thermal stability of SR/m-POSS composites becomes better, while the tensile strength decreases. SR/m-POSS (100/1.5) composite not only exhibits excellent thermal stability, but also retains good mechanical properties, with a tensile strength of 9.2 MPa and elongation at a break of 587%. Therefore, this study provides an effective strategy for preparing SR composites with excellent mechanical and thermal properties, significantly expanding the application of silicone rubber in fields such as aerospace sealing materials and high-voltage wiring harnesses for new energy vehicles.

## Figures and Tables

**Table 2:** Statistical comparison of mechanical properties of the composites.

Sample	Tensile Strength (MPa)	*p*-Value vs. SR/1.5POSS	Elongation at Break (%)	*p*-Value vs. SR/1.5POSS
SR	9.9 ± 0.4	- ^a^	540 ± 28	- ^a^
SR/1.5POSS	5.8 ± 0.4	Reference	224 ± 18	Reference
SR/1.5m-POSS	9.2 ± 0.3	*** (*p* < 0.001)	587 ± 23	*** (*p* < 0.001)

1. Statistical significance was defined as: *** *p* < 0.001. 2. - ^a^: Comparison with the reference group (SR/1.5POSS) was not performed. 3. All values are reported as mean ± standard deviation (n = 5). Statistical comparisons were performed using an unpaired two-sample Student’s *t*-test.

**Table 3:** Comparison of mechanical properties between the prepared SR composites and previously reported SR composites.

Material	Filler	Content (phr)	Tensile Strength (MPa)	Elongation at Break (%)	Ref.
SR (SiO_2_)	POSS	1.5	5.8	223	This study
SR (SiO_2_)	m-POSS	1.5	9.2	587	This study
SR (SiO_2_)	POSS/CNT	0.1/1.0	10.2	456	[22]
SR (SiO_2_)	ZIF-67	0.5	5.7	273	[5]
SR (SiO_2_)	CNTs@Fe_2_O_3_	3	6.0	450	[4]

## Data Availability

Data are contained within the article.
